# Rolling Bearing Fault Diagnosis Based on SABO–VMD and WMH–KNN

**DOI:** 10.3390/s24155003

**Published:** 2024-08-02

**Authors:** Guangxing Liu, Yihao Ma, Na Wang

**Affiliations:** 1 School of Electronic Engineering, Xi’an Shiyou University, Xi’an 710065, China; gxliu@xsyu.edu.cn (G.L.); 22211030370@stumail.xsyu.edu.cn (N.W.); 2 Key Laboratory of Measurement and Control Technology for Oil and Gas Wells, Xi’an Shiyou University, Xi’an 710065, China

**Keywords:** bearing, fault diagnosis, variational mode decomposition (VMD), WMH-K nearest neighbor (KNN), subtraction average-based optimizer (SABO)

## Abstract

To improve the performance of roller bearing fault diagnosis, this paper proposes an algorithm based on subtraction average-based optimizer (SABO), variational mode decomposition (VMD), and weighted Manhattan-K nearest neighbor (WMH–KNN). Initially, the SABO algorithm uses a composite objective function, including permutation entropy and mutual information entropy, to optimize the input parameters of VMD. Subsequently, the optimized VMD is used to decompose the signal to obtain the optimal decomposition characteristics and the corresponding intrinsic mode function (IMF). Finally, the weighted Manhattan function (WMH) is used to enhance the classification distance of the KNN algorithm, and WMH–KNN is used for fault diagnosis based on the optimized IMF features. The performance of the SABO–VMD and WMH–KNN models is verified through two experimental cases and compared with traditional methods. The results show that the accuracy of motor-bearing fault diagnosis is significantly improved, reaching 97.22% in Dataset 1, 98.33% in Dataset 2, and 99.2% in Dataset 3. Compared with traditional methods, the proposed method significantly reduces the false positive rate.

## 1. Introduction

The electric oil drilling rig plays a vital and irreplaceable role in the petroleum industry, serving as a cornerstone of oil exploration. Within this critical equipment, rolling bearings hold a position of particular importance as they support and sustain the stable functioning of the rotating machinery components. Nevertheless, the continuous operation under heavy loads, high temperatures, and other influential factors often renders these rolling bearings susceptible to failure, thus posing a significant risk to their lifespan [1,2]. Consequently, the prompt and precise diagnosis of rolling bearing faults in electric drilling rigs holds paramount importance in safeguarding the secure operation of production equipment [3].

In recent years, vibration signal analysis has emerged as a prevalent approach for diagnosing faults in roller bearings. MMM Islam et al. [4] proposed a robust multiple-combination fault diagnosis framework employing an equalization function model for rolling bearings, along with an enhanced single-correlation support vector machines (OAA-MCSVM) classifier. Tan Chao et al. [5] introduced a hybrid framework based on multienvelope teaching optimization (METLBO), integrating parameter-optimized variational mode decomposition (VMD) with an improved support vector machine (ISVM). Zhuang Deyu et al. [6] developed a feature extraction technique employing VMD and sample entropy, accompanied by a refined sequential minimization algorithm using optimal parameters for fault identification. Luo Jianqing et al. [7] proposed a novel rolling bearing fault diagnosis approach, amalgamating adaptive VMD and SR via an improved differential search (IDS) optimization. Ma Jinghua et al. [8] presented a rolling bearing fault diagnosis method integrating an improved VMD adaptive wavelet threshold with noise reduction. Zhenya Quan et al. [9] employed MDE to isolate strong background noise amid weak fault features of rolling bearings, constructing a multi-label k-nearest neighbor (ML-KNN) classifier for recognizing patterns associated with the subtle faults of rolling bearings. Ali Dibaj et al. [10] employed an end-to-end finetuning approach for VMD, a convolutional neural network (CNN), and a novel fault classification scheme to diagnose both single and compound faults in automobile gearbox systems with varying degrees of fault severity. Yuxing Li et al. [11] proposed a variable step size multi-scale single threshold SloEn (VSM-StSloEn), which can not only reflect the complexity of information hidden in different time scales but also make up for the shortcomings of traditional multi-scale processing. Wang Yaping et al. [12] proposed a rolling bearing fault diagnosis method based on the whale optimization algorithm–variational mode decomposition (WOA–VMD) and Graph Attention Network (GAT), utilizing the KNN method to construct graph-structured data. Yuxing Li et al. [13] developed a multivariate SloEn (mvSloEn) and extended it to multi-scale mvSloEn (mvMSloEn), which not only accounts for the correlation of time series complexity within and across channels but also mirrors the complexity of multi-channel time series over multiple scales. While most traditional optimization algorithms mentioned above excel in parameter optimization and fault classification, the optimization algorithms themselves are faced with certain challenges, including slow optimization speeds and susceptibility to local optima, thereby diminishing the accuracy of fault diagnosis. Based on the aforementioned research, this paper employs the subtraction average-based optimizer (SABO) optimization algorithm, known for its straightforward principles and effective optimization outcomes.

Traditional fault diagnosis algorithms exhibit certain limitations in handling fault diagnosis. Lei, Xue et al. [14] and Jiang, Haisheng et al. [15] employed Extreme Learning Machines (ELMs) for fault classification. However, the significant impact of the hidden layer often results in unstable classification performance. Yiyao et al. [16] utilized Long Short-Term Memory (LSTM) for fault classification, but the limitations of a single model can lead to overfitting issues with samples. Building upon this, Guo, Yurong, et al. [17] employed convolutional neural networks (CNNs) and Bidirectional LSTM (BiLSTM) for bearing fault classification. However, the drawback of a lengthy training time arises due to the abundance of fault samples. Zhang, Mei et al. [18] utilized support vector machines (SVMs) to avoid prolonged training times, but the diverse nature of faulty datasets makes it challenging for SVM to find an ideal hyperplane. Kumar, HS et al. [19] used the KNN classifier to address this issue. Nevertheless, since each fault has a different prominent effect, the diagnostic performance for similar fault types may not be ideal. Sun Dingyi and others used a physics-inspired multi-modal machine learning and rotating machinery fault diagnosis based on adaptive correlation fusion [20], but it was not successful enough for multi-source signal processing. Al-Haddad, Luttfi A, et al. used embedded recorded data and stacked machine learning models to fault-diagnose drone actuator damage [21], but the training time was too long for complex application scenarios. Therefore, this paper adopts the weighted Manhattan function (WMH). While considering the Manhattan distance, the WMH introduces weights for each fault type, optimizing the KNN classifier to achieve better performance by accounting for the specificity of each fault. VMD can adaptively decompose signals into several intrinsic mode functions (IMFs) without presetting the number of decomposition layers. This feature makes VMD more flexible and efficient when processing complex signals. VMD can effectively separate signals with different frequency components into different IMFs, thus avoiding the modal aliasing problem. This is of great significance for accurately extracting signal features, especially in vibration signal analysis. VMD performs well when processing noisy signals and can effectively extract useful information from the signal and suppress the noise. This makes VMD highly robust and reliable in practical applications. WMH–KNN combines the weighted Manhattan distance and the KNN classifier and is able to introduce the weight of each fault type while considering the Manhattan distance, thereby optimizing the performance of the KNN classifier. This weighting strategy makes the classifier more flexible and accurate when dealing with different types of faults. By introducing weights, WMH can better reflect the importance of different fault characteristics and improve the classification performance of the classifier on complex multi-modal data. KNN itself has a good performance in processing high-dimensional space data. Combined with the weighted Manhattan distance, WMH–KNN can more effectively handle complex data in high-dimensional space and improve classification accuracy. The SABO–VMD–WMH–KNN model is particularly suitable for multi-modal problems and data processing in high-dimensional spaces and can provide powerful solutions. Its superiority is that in the process of optimizing VMD parameters in the fault diagnosis system, the search speed is significantly improved and the dilemma of sub-optimal solutions is avoided, thereby providing strong support for the accuracy and reliability of fault diagnosis. This model performs well in the analysis of motor-bearing vibration signals and can effectively extract relevant information from vibration signals to improve the accuracy and reliability of fault diagnosis. By comprehensively utilizing SABO, VMD and WMH–KNN methods, a fault diagnosis system with high accuracy and high reliability is established. Its superiority lies in the process of optimizing VMD parameters in the fault diagnosis system, which significantly improves the search speed and avoids falling into the dilemma of sub-optimal solutions, thus providing strong support for accurate and reliable fault diagnosis. A motor-bearing fault diagnosis model termed SABO–VMD–WMH–KNN is established. SABO optimizes VMD parameters to extract optimal modal information from vibration signals, while the WMH–KNN method combines the weighted Manhattan distance with KNN machine learning for effective feature extraction and classification. Together, they form a comprehensive fault diagnosis model. This model is designed to effectively extract pertinent information from motor-bearing vibration signals, thereby enhancing the precision and dependability of fault diagnosis.

The method proposed in this article significantly advances existing technology by introducing the SABO algorithm to enhance the accuracy of VMD parameter optimization and employs the weighted Mahalanobis distance (WMH) to improve the classification performance of the KNN algorithm. This innovation markedly improves accuracy and robustness in complex mechanical fault diagnosis. Not only does it overcome the limitations of traditional methods, but it also demonstrates superior performance in experiments, indicating its broad potential and value in practical industrial applications. The research results offer new insights and technical support for machine fault diagnosis, highlighting significant economic benefits in improving equipment maintenance efficiency and reducing downtime.

The structural arrangement is as follows: Section 2 will introduce the theoretical basis, including VMD, SABO, and WMH–KNN. Section 3 will describe the research methods used in this study. Section 4 will present and discuss the results from Dataset 1 in detail. Section 5 will present and discuss the results from Dataset 2. Finally, Section 6 will summarize the research conclusions, discuss the limitations of the study, and provide suggestions for future research directions.

## 2. Theoretical Basis

### 2.1. Principle of VMD Algorithm

VMD, as highlighted by [22], represents a fully non-recursive adaptive signal decomposition technique utilized for breaking down a signal or dataset into numerous local oscillation modes. Each of these modes corresponds to the local frequency and amplitude of the signal. This particular decomposition characteristic renders VMD well-suited for analyzing non-stationary signals, given its capability to capture transient occurrences and local frequency fluctuations within the signal. The mathematical depiction is as follows:

(1) $$\begin{array}{c} u_{k}\left(t\right)=A_{k}\left(t\right){\cos\varphi }_{k}\left(t\right) \end{array}$$

In Formula (1), $u_{k}\left(t\right)$ represents the kth modalunction, which satisfies Formula (2), $A_{k}\left(t\right)$ represents the instantaneous amplitude, and $\varphi_{k}\left(t\right)$ denotes the instantaneous phase.

(2) $$\begin{array}{c} x\left(t\right)=\sum_{k=1}^{n}u_{k}\left(t\right) \end{array}$$

In Formula (2), $x\left(t\right)$ is the given continuous signal.

Post VMD decomposition, a sequence of local oscillation modes, denoted as $u_{k}\left(t\right)$, is acquired, with each mode representing a localized feature of the signal [23]. These modes are instrumental in analyzing various aspects, such as the frequency spectrum, transient components, oscillatory characteristics, and more. Among these, the intrinsic mode function (IMF) component features a restricted bandwidth, with its central frequency designated as $\omega_{k}$, and the spectrum displaying sparsity. By formulating the constraint problem, the resulting constrained variational model and constraint model equations are as follows:

(3) $$\begin{array}{c} \left\{\begin{array}{c} \min\left\{u_{k}\right\},\left[\omega_{k}\right]\left\{\sum_{k}∥\partial_{t}\left[\left(\delta \left(t\right)+\frac{j}{\pi t}\right)*u_{k}\left(t\right)\right]e^{-j\omega_{k}t}{∥}_{2}^{2}\right\}\\ s.t.\sum_{k}u_{k}\left(t\right)=f\left(t\right) \end{array}\right. \end{array}$$

In Formula (3), $u_{k}$ is IMF components, $\omega_{k}$ is frequency centers, $\delta \left(t\right)$ is a Dirac function, $\partial_{t}$ is the partial derivative with respect to time *t*, and $f\left(t\right)$ is the original signal. Then, the model is solved, and the penalty factor α and Lagrange multiplication operator λ are introduced to transform the constrained variational problem into an unconstrained variational problem [24]. The augmented Lagrange expression is obtained as follows:

(4) $$\left\{\begin{array}{c} \begin{array}{c} A=\alpha \sum_{K}||\partial_{t}\left[\left(\delta \left(t\right)+\frac{j}{\pi t}\right)*u_{k}\left(t\right)\right]e^{-j\omega_{k}t}|{|}_{2}^{2}\\ B=||f\left(t\right)-\sum_{k}u_{k}\left(t\right)|{|}_{2}^{2}\\ C=\left\langle \lambda \left(t\right),f\left(t\right)-\sum_{k}u_{k}\left(t\right)|\right\rangle \\ L\left(\left\{u_{k}\right\},\left\{\omega_{k}\right\},\lambda \right)=A+B+C \end{array} \end{array}\right.$$

In Formula (4), the central frequency and bandwidth of each IMF are constantly updated by the alternating multiplier direction algorithm, and the saddle point of Equation (4) is found as the solution of Equation (3) [25].

### 2.2. Principle of SABO Algorithm

The fundamental concept behind the SABO involves the mathematical notion of subtracting the average information from the algorithm’s search agent [26]. SABO boasts several advantages, including a potent optimization capability, rapid convergence, robustness, and commendable stability.

From a mathematical standpoint, Formula (5) allows for the representation of the algorithm’s population using a matrix. The primary position of the search agent within the search space is randomly initialized, as described by Formula (6).

(5) $$\begin{array}{c} \begin{array}{c} X=\left[\begin{array}{c} X_{1}\\ \vdots \\ X_{i}\\ \vdots \\ X_{N} \end{array}\right]=\left[\begin{array}{ccccc} x_{1,1} & \cdots & x_{1,d} & \cdots & x_{1,m}\\ \vdots & \ddots & \vdots & \vdots & \vdots \\ x_{i,1} & \cdots & x_{i,d} & \cdots & x_{i,m}\\ \vdots & \vdots & \vdots & \ddots & \vdots \\ x_{N,1} & \cdots & x_{N,d} & \cdots & x_{N,m} \end{array}\right] \end{array} \end{array}$$

(6) $$\begin{array}{c} \begin{array}{c} \begin{array}{c} x_{i,d}=lb_{d}+r_{i,d}\cdot \left(ub_{d}-lb_{d}\right)\\ i=1,\ldots ,N,d=1,\ldots ,m\# \end{array}\# \end{array} \end{array}$$

In Formulas (5) and (6), X represents the SABO population matrix, $X_{i}$ signifies the search agent in the algorithm, $x_{i,d}$ denotes the value of the decision variable of the d th dimension of the i th search agent in the search space, N represents the number of search agents, m indicates the number of decision variables, and $r_{i,d}$ is a random number within the range of [0, 1], while $lb_{d}$ and $ub_{d}$ represent the lower and upper bounds of the d th decision variable, respectively.

The SABO algorithm introduces a new computing concept “-_v”, which is called the v-subtraction of search agent B and search agent a, which is defined as follows:

(7) $$\begin{array}{c} A{-}_{v}B=sign\left(F\left(A\right)-F\left(B\right)\right)\left(A-\vec{v}*B\right) \end{array}$$

In Equation (7), $\vec{v}$ is a vector with the dimension of m, where the components are random numbers generated from the set {1,2}. The operator represents the Hadamard product of two vectors. And $F\left(A\right)$ and $F\left(B\right)$ represent the target function values of the search agent A and B, respectively; sign is the symbolic function.

In the SABO algorithm, the displacement of any search agent $X_{i}$ in the search space is calculated by the arithmetic mean of the ${-}_{v}$ subtraction of each search agent $X_{j}$. The location is updated as follows:

(8) $$\begin{array}{c} X_{i}^{new}=X_{i}+{\vec{r}}_{i}*\frac{1}{N}\sum_{j=1}^{N}\left(\begin{array}{ccc} X_{i} & {-}_{v} & X_{j} \end{array}\right),i=1,2,\ldots ,N \end{array}$$

In Formula (8), $X_{i}^{new}\;$ represents the newly proposed location of the ith search agent, signifying the updated position of search agent $X_{i}$. N is the total number of search agents, representing the complete set of agents or individuals engaged in the search process. ${\vec{r}}_{i}$ is a vector with a dimension of m, where each component is a normal distribution value drawn from the interval [0, 1].

The replacement formula for particle position is as follows:

(9) $$\begin{array}{c} X_{i}=\left\{\begin{array}{c} X_{i}^{new},F_{i}^{new}<F_{i}\\ X_{i},else \end{array}\right. \end{array}$$

**Figure 1:**
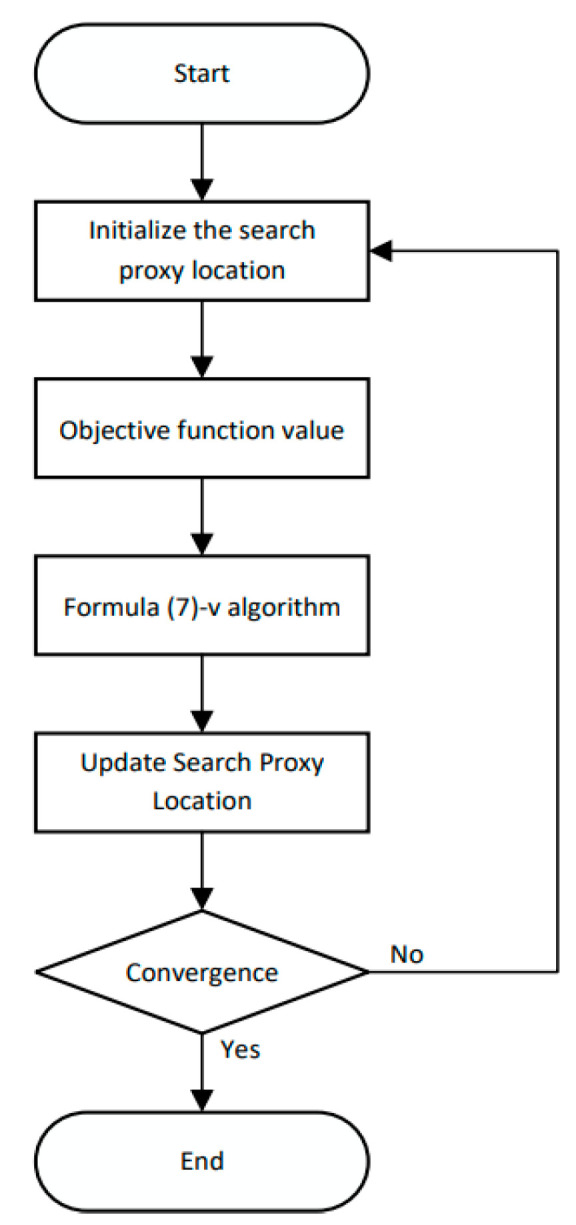
SABO algorithm flow chart.

In Equation (9), $F_{i}$ represents the target function value of the search agent $X_{i}$ and $F_{i}^{new}$ represents the target function value of the search agent $X_{i}^{new}$. The specific flow chart of the algorithm is shown in Figure 1.

### 2.3. WMH–KNN Algorithm Principle

The KNN method is extensively employed in pattern classification due to its simplicity [27]. The basic idea involves observing the categories of the K samples in the sample space that are closest in distance to the sample to be classified. The category that prevails among these K samples is then assigned to the sample to be classified. The distance calculation formula between samples is as follows:

(10) $$\begin{array}{c} D_{i}=\sum_{j=1}^{m}\;\left|x_{ij}-y_{ij}\right|\cdot w_{j} \end{array}$$

In Formula (10), assuming there are two sample matrices, X and Y, where $x_{ij}$ and $y_{ij}$ respectively represent the elements in X and Y, n is the number of samples, m is the number of features, and the feature weight vector is denoted as $W=[w_{1},w_{2},...,w_{m}]$, where $w_{j}$ is the weight of the jth feature.

Determine the nearest neighbor parameter c and find the c-nearest neighbors to the sample to be classified. The formula is as follows:

(11) $$\begin{array}{c} c={\mathrm{argmax}}_{i}D_{i} \end{array}$$

In Formula (11), ${\mathrm{argmax}}_{i}$ is the nearest neighbor sample.

The classification sample is as follows:

(12) $$\begin{array}{c} \hat{y}={\mathrm{argmax}}_{c}\sum_{i=1}^{K}\;I\left(y_{i}=c\right) \end{array}$$

In Formula (12), $\hat{y}$ is the category of the sample to be classified, $y_{i}$ is the category of the i-th training sample closest to the sample to be classified, $I\left(y_{i}=c\right)$ is the indicator function, if $y_{i}=c$, it is 1, otherwise it is 0.

The computational complexity of the WMH–KNN algorithm is mainly affected by the following factors: First, the KNN algorithm itself needs to calculate the distance between the sample to be classified and all training samples. The complexity of this process increases with the size of the training dataset and the increase in feature dimensions. Second, the calculation of the Mahalanobis distance involves the inversion of the covariance matrix, which will increase the computational complexity in high-dimensional feature space. In addition, the Hamming distance is used to process discrete features, and its computational complexity is relatively low, but it will also increase a certain computational burden when performing comprehensive weighted calculations. In general, the WMH–KNN algorithm has a high computational complexity when processing large-scale datasets and high-dimensional feature spaces. It is necessary to comprehensively consider the optimization of the dataset size, feature dimension, and calculation steps to ensure the efficiency and practicality of the algorithm.

## 3. Diagnosis Model Based on SABO–VMD–WMH–KNN

### 3.1. SABO–VMD Model

The SABO algorithm is harnessed for the optimization of the parameters α and k in the VMD process. By employing the composite index of permutation entropy and mutual information entropy as the fitness function, the fitness function is set to the minimum, and the SABO algorithm is used to find parameters. Through continuous iteration and comparison of fitness function values, the minimum value is found to terminate the iteration and obtain the corresponding k and α; that is, the optimal parameters. In conjunction with permutation entropy and mutual information entropy, the performance of VMD can be more comprehensively assessed. This approach enhances the adaptability of the optimization process [28].

The calculating formula of permutation entropy ($P_{e}$) is as follows:

(13) $$\begin{array}{c} P_{e}=\frac{\left(-\sum_{i=1}^{k}P_{i}\cdot \mathrm{lg}\left(P_{i}\right)\right)}{\mathrm{lg}\left(m!\right)} \end{array}$$

In Formula (13), k represents the number of different permutations, $P_{i}$ represents the relative frequency of each arrangement, and m represents the length of the subsequence.

The permutation entropy can effectively reflect the complexity of the time series, and the permutation entropy can better reflect the regular degree of the time series after normalization [29]. The smaller the permutation entropy, the more regular the time series; on the contrary, the stronger the randomness of the time series.

The calculating formula of mutual information entropy (${MI}_{e}$) is as follows:

(14) $$\begin{array}{c} \begin{array}{cc} {MI}_{e} & =- \end{array}\sum_{i=1}^{N}p_{i}\mathrm{lg}p_{i} \end{array}$$

In Formula (14), $p_{i}$ represents the relative frequency of each arrangement.

Mutual information entropy serves as a concept utilized to quantify the extent of system uncertainty or information disarray, commonly employed within the realms of information theory and signal processing [30]. It is typically leveraged to assess the order or disorder present within a signal. In the context of bearing failure, the operational process generates periodic impacts, leading to a more organized signal. This sense of order is often manifested in the reduction in information entropy. Consequently, by scrutinizing the information entropy or mutual information entropy of the signal, it becomes possible to detect any faults or anomalies within the bearing. Such changes in the signal’s order can be employed to diagnose issues and implement appropriate maintenance measures [31].

Permutation entropy and mutual information entropy are amalgamated by establishing a composite index, serving as the fitness function during the optimization process. This composite index takes shape as a weighted combination of permutation entropy and mutual information entropy. The formula for calculating the composite index is as follows:

(15) $$\begin{array}{c} H=\omega_{1}\cdot P_{e}+\omega_{2}\cdot {MI}_{e} \end{array}$$

In Formula (15), $\omega_{1}$ and $\omega_{2}$ are the weights of permutation entropy and mutual information entropy, where $\omega_{1}=\omega_{2}=0.5$. Combining permutation entropy and mutual information entropy as the fitness function to optimize VMD parameters can use the complementarity of the two to improve the comprehensiveness of signal feature extraction and the accuracy of fault detection, thereby achieving better optimization results. The combined fitness function has stronger adaptability to different types of signals and can show better robustness under various complex working conditions. By comprehensively evaluating the complexity of the signal and the degree of information sharing, the error caused by a single method can be reduced, and the overall optimization effect can be improved. This method is not only suitable for a single type of signal analysis but can also be applied to a variety of signal types and application scenarios with wide applicability.

The main task of the VMD algorithm is to decompose the signal into several intrinsic mode functions (IMFs). The number of decomposed modes K has a direct impact on the computational complexity. Generally speaking, the more decomposed modes, the greater the computational effort. VMD requires multiple iterations to converge to a stable solution. The computational steps involved in each iteration include frequency domain transformation and optimization of the signal, and the complexity of these steps is usually determined by the number of signal samples. SABO requires multiple calculations in each iteration to update the position of the search agent. This includes calculating the objective function value, updating the position, and performing weighted calculations. The complexity of each iteration is determined by the number of search agents and the dimensionality of the decision variables. The computational complexity of SABO–VMD is limited by the complexity of VMD and SABO. In practical applications, factors such as the signal length, number of modes, number of optimization iterations, and number of search agents need to be considered.

**Figure 2:**
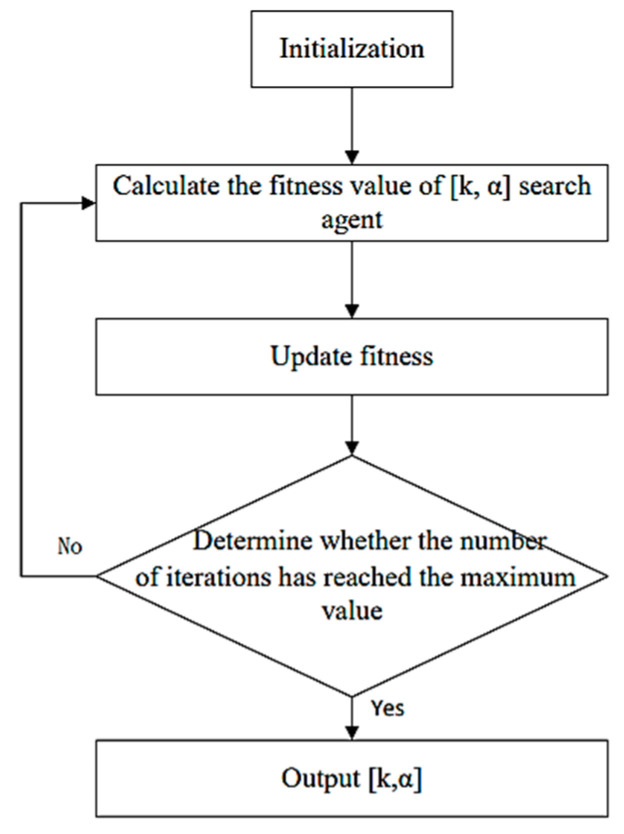
SABO–VMD optimization model.

The steps and process of the SABO–VMD prediction model are shown in Figure 2:

The specific steps for SABO to optimize VMD parameters are as follows:

Parameter initialization. For example, the number of populations, X, the number of search agent, N dimensions, d, the optimization range of parameters, etc.

Compute the Nth fitness value corresponding to each search agent’s position, and, subsequently, determine the minimum value of the composite index as the fitness function, integrating both the compound index permutation entropy and mutual information entropy.

Update the particle position according to fitness and use Formula (9) to update.

Determine whether the algorithm reaches the maximum number of iterations; if so, the loop ends and outputs the optimal SABO location [k,α] and optimal fitness value; if not, return to Step 2.

### 3.2. SABO–VMD–WMH–KNN Model

After obtaining the IMF components through the SABO–VMD model, the statistical characteristics (mean, standard deviation, kurtosis, and skewness) of each IMF component are calculated. The statistical characteristics can reflect the distribution characteristics and fluctuations of the signal on the frequency scale. These statistical characteristics contain important time–frequency characteristics and information about the signal and can better describe the overall characteristics of the signal.

Suppose the original signal is x(t), and the IMF components decomposed by the SABO–VMD model are where *i* = 1, 2,..., *n*, and *n* is the number of IMF components. For each IMF component $c_{i}(t)$, the following statistical characteristics can be calculated:

Mean ($u_{i}$)

(16) $$\begin{array}{c} u_{i}=(1/T)*\int c_{i}(t)dt \end{array}$$

Standard Deviation ($\sigma_{i}$)

(17) $$\begin{array}{c} \sigma_{i}=\sqrt{\left[(1/T)*\int {\left(c_{i}(t)-u_{i}\right)}^{2}dt\right]} \end{array}$$

kurtosis ($k_{i}$)

(18) $$\begin{array}{c} k_{i}=(1/T)*\int {\left(c_{i}(t)-u_{i}\right)}^{4}dt/{\sigma_{i}}^{4} \end{array}$$

skewness ($\gamma_{i}$)

(19) $$\begin{array}{c} \gamma_{i}=(1/T)*\int {\left(c_{i}(t)-u_{i}\right)}^{3}dt/{\sigma_{i}}^{3} \end{array}$$

where *T* is the total time length of the signal.

These statistical features are used as input features to form a feature vector, which can be input into the WMH–KNN model for training and diagnosis. The mean describes the average level of the signal at this frequency scale, which helps to reflect the overall trend of the signal. The standard deviation reflects the degree of fluctuation of the signal at this frequency scale, which helps to identify abnormal fluctuations. Kurtosis and skewness describe the distribution shape of the signal at this frequency scale, which helps to discover the non-Gaussian characteristics of the signal. Combining these statistical features, the time–frequency characteristics of the signal at different frequency scales can be fully characterized, providing valuable input features for subsequent WMH–KNN model training and diagnosis. These features are simple and easy to calculate and have low computational complexity during model training and inference. Compared with directly using the original signal, the statistical features are more robust and less susceptible to noise interference. This method can effectively extract the time–frequency characteristic information of the signal at different frequency scales, providing information-rich input features for subsequent model applications.

**Figure 3:**
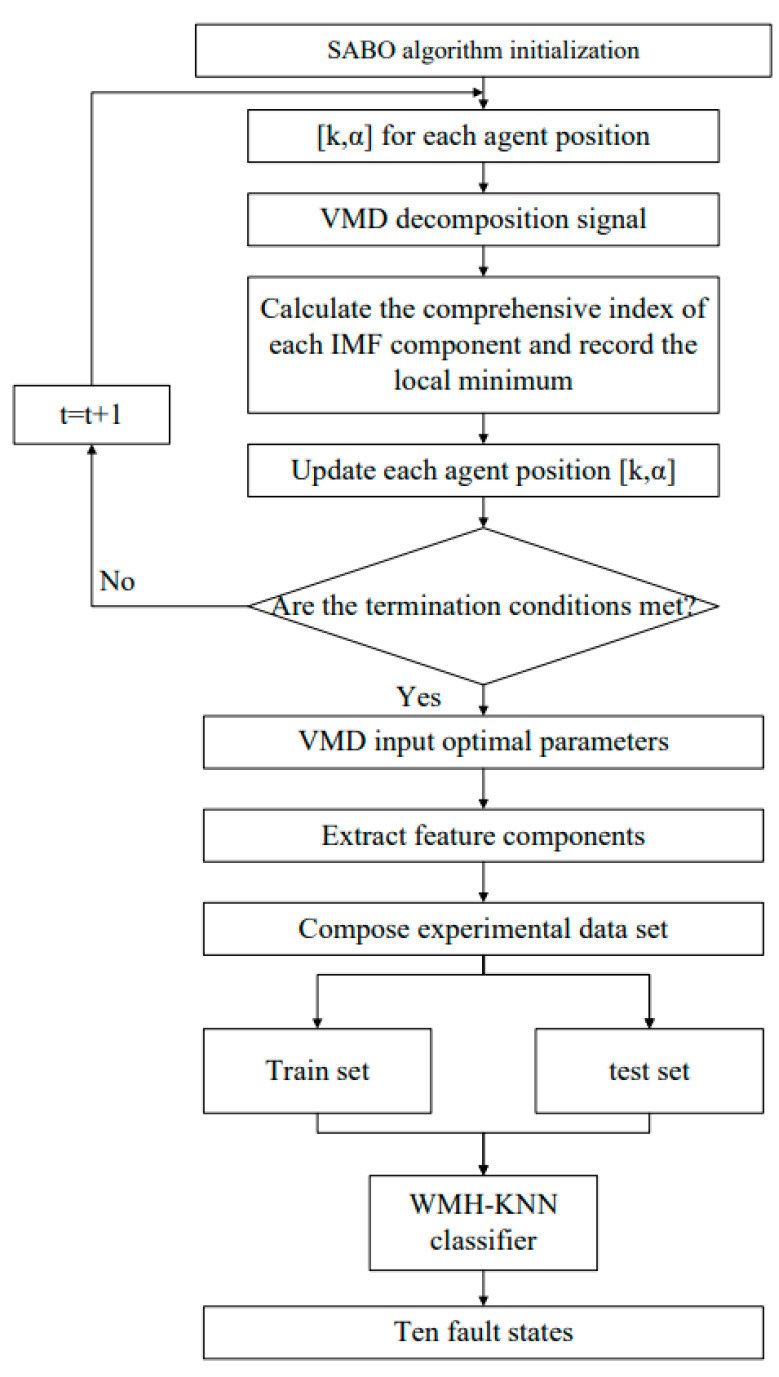
SABO–VMD–WMH–KNN bearing fault diagnosis model.

Drawing from the theoretical foundations of the aforementioned algorithms, this paper introduces a motor-bearing diagnosis approach based on SABO–VMD–WMH–KNN. The fault diagnosis process is depicted in Figure 3.

The specific flow of the SABO–VMD–WMH–KNN diagnostic model is as follows:

Gather fault signals corresponding to 10 different states. These signals serve as the input data for the diagnostic model.

Combine permutation entropy and mutual information entropy to form a compound index. This composite index is used to evaluate the effectiveness of the VMD parameters. Apply the SABO algorithm to optimize the variational mode decomposition (VMD) parameters, denoted as ([k, α]). Here, k is the number of intrinsic mode functions (IMFs), and α is the balancing parameter.

Utilize the principle of minimum compound index entropy and mutual information entropy to evaluate and select the best IMF component from each of the 10 states based on the minimum value of the compound index. This results in 10 optimal IMF components, one for each state.

Use the 10 selected IMF components as feature vectors. These feature vectors capture the essential characteristics of the fault signals for each state.

Train the WMH–KNN model using the feature vectors derived from the selected IMF components. Perform fault diagnosis using the trained WMH–KNN model. This model classifies new fault signals into one of the 10 states based on the trained feature vectors.

The pseudo-code of the SABO–VMD–WMH–KNN Algorithm 1 is as follows:
**Algorithm 1:** [26] SABO–VMD–WMH–KNN**Input**: Original signal ***X***. K, *α*, search agents *N*, number of iterations *T*, Number of neighbors *K*, weighting coefficients *W*. **Output**: classified labels ***Y***1: Function VMD(***X***, *K*, *α*)2: *U*, *K*//Initialize matrix *U* with *K* rows and length(x) columns3: **for**
*i* = 1:*K*4: *U*(*i*, :)//Initialize mode *U*(*i*, :) with random values5: **return** *U*//Update mode *U*(*i*, :)6: Function SABO(U, N, T)7: **for**
*i* = 1:*T*8:   **for**
*i* = 1:*N*9: *F*(*i*), *X*(*i*, :)//Calculate objective function *F*(*i*) for search agent *X*(*i*, :)10:   **for**
*i* = 1:*N*11: Compute mean displacement based on other agents12: **return** *U*13: Function WMH_KNN(U, TrainingData, K, Weights)14: **for**
*i* = 1:TestData15: W*MH//Compute WMH distance to all training samples16: K//Voting categories17: **return *Y***

The following is a discussion of the sensitivity of each component parameter:

SABO–VMD:

Number of search agents: The number of search agents directly affects the search range of the optimization process and the accuracy of the results. More search agents can improve the accuracy of the results but increase the computational complexity.

Number of iterations: The number of iterations determines the depth of the optimization process. Too few iterations may lead to insufficient convergence, while too many iterations will increase the computational time.

Number of decomposition patterns (K): The number of decomposition patterns affects the fineness of signal decomposition. More patterns can capture more subtle signal features but also increase the amount of computation and complexity.

Penalty parameter: The penalty parameter is used to balance the degree of data fitting and the degree of smoothness of the decomposition results. Different penalty parameter settings will affect the quality of the decomposition results.

WMH–KNN:

K value: The K value determines the number of neighbors selected in the KNN algorithm. A larger K value can improve the stability of the classification but may reduce the accuracy of the classification.

Weighting coefficient: The weighting coefficient of the Mahalanobis distance and the Hamming distance affects the comprehensive effect of the distance metric. Different weighting coefficients will affect the accuracy and robustness of the classification results.

## 4. Experimental Case Analysis 1

### 4.1. Dataset Collection 1

**Figure 4:**
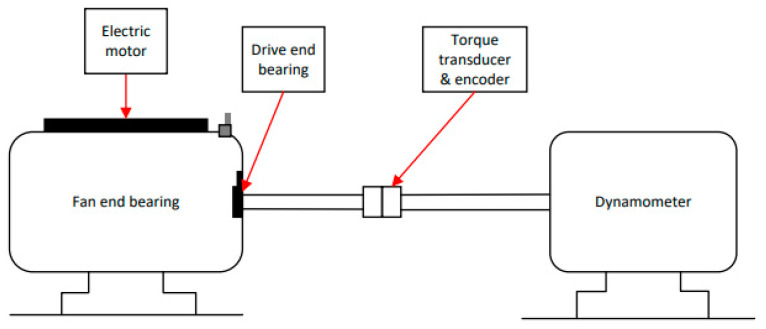
Case Western Reserve University bearing data collection platform.

The faulty Dataset 1 comes from the bearing data acquisition experimental platform of Case Western Reserve University. The experimental platform consists of a two-horsepower motor, a torque encoder, a power tester, and an electronic controller. The experimental platform is shown in Figure 4.

Set the sliding window to 1000; the number of fault sample points of each data is 2048, and the sample size of each fault type is 120. After all the data sliding window is finished, it is integrated into a dataset, and the corresponding labels of the ten states are 1 to 10. 

Then, each data is labeled; Table 1 is an introduction to the bearing dataset. In Table 1, the bearing to be tested supports the shaft of the motor. The drive end bearing is SKF6205, the sampling frequencies are 12 KHz and 48 kHz, the fan end bearing is SKF6203, and the sampling frequency is 12 KHz. In this experiment, the bearing data of the drive end is selected, and the sampling frequency is 12 KHz. There are ten states, namely, the normal state (marked T here); the inner ring fault when the diameter is 0.007 inches, and the speed is 1750 (marked IF-7 here); the rolling body fault when the diameter is 0.007, and the speed is 1750 (marked RF-7 here); the outer ring fault when the diameter is 0.007, and the speed is 1750 (marked OF-7 here); inner ring failure when the diameter is 0.014, and the speed is 1750 (marked IF-14 here); roller failure when the diameter is 0.014, and the speed is 1750 (marked RF-14 here); outer ring failure when the diameter is 0.014, and the speed is 1750 (marked RF-14 here); inner ring failure when the diameter is 0.021, and the speed is 1750 (marked IF-21 here); roller failure when the diameter is 0.021, and the speed is 1750 (marked RF-21 here) and outer ring failure when the diameter is 0.021, and the speed is 1750 (marked OF-21 here).

### 4.2. Optimization Algorithm Model Comparison Results

**Figure 5:**
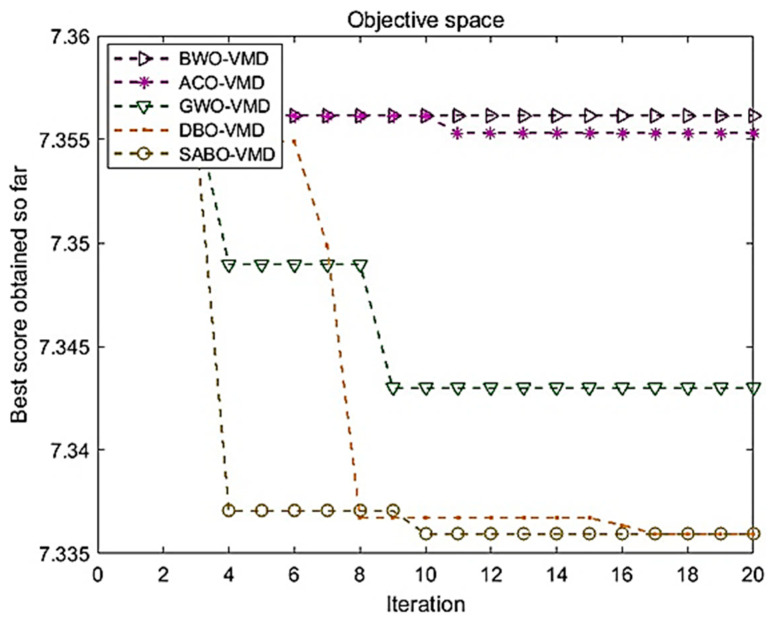
Fitness curves of five optimization algorithms.

There are ten fault states in this experiment, with the simulation platform being MATLAB 2019b. The analysis involves five optimization algorithms, namely, the Ant Colony Algorithm (ACO), Grey Wolf Algorithm (GWO), Beluga Algorithm (BWO), Dung Beetle Algorithm (DBO), and the SABO as utilized in this study. The optimization results of the VMD parameters are obtained by employing the composite index permutation entropy and mutual information entropy as the fitness function. Figure 5 illustrates the fitness curves of the aforementioned five algorithms.

It is evident from Figure 4 that SABO yields the most favorable results in optimizing the VMD parameters, achieving the minimum objective function in the tenth iteration. The *X*-axis represents the number of iterations in the optimization process. Each iteration corresponds to an optimization calculation, and the VMD parameters are gradually adjusted to achieve the optimal result. The *Y*-axis represents the value of the objective function. The objective function is an evaluation criterion in the optimization process, usually reflecting the error or loss value. The smaller the objective function value, the more optimized the model parameters are. This outcome marks the superior performance of the SABO model in comparison to the other four optimization algorithms.

### 4.3. Model Optimization Result

The main parameters of VMD are k and α [30]. Whether the vibration signal decomposition process is completed or not depends on these two main input parameters. The VMD parameters of each fault state are optimized through SABO and the optimal parameters k and α of VMD decomposition of each fault state are obtained by combining entropy H as the optimization index, as shown in Table 2. For the IMF number k, a smaller k results in the inability to completely decompose all useful information in the signal, affecting the accuracy of fault diagnosis. Larger k introduces too much noise and redundant information, increases computational complexity, and may affect the generalization ability of the model. For the equilibrium parameter α, a smaller α causes the decomposed IMF to contain too much noise, which affects the extraction of signal features. The IMF decomposed by a larger α is too smooth and may lose some important detailed information. Decompose the fault signal through VMD and obtain several IMF components. A composite metric (combination of permutation entropy and mutual information entropy) is used to select optimal IMF components. Finally, the best IMF component in each state is selected, and a total of 10 IMF components are used as feature vectors.

**Figure 6:**
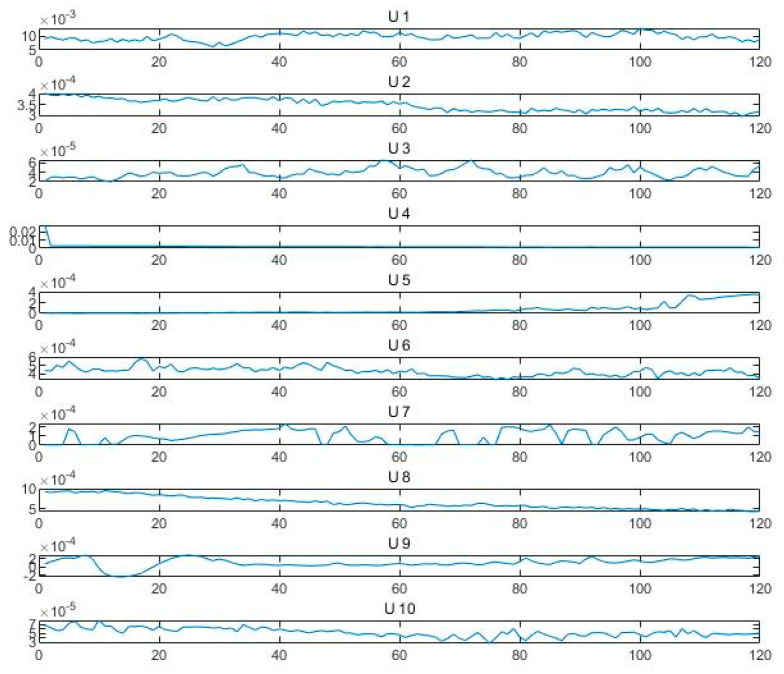
The mean eigenvector of the best IMF for each fault state.

**Figure 7:**
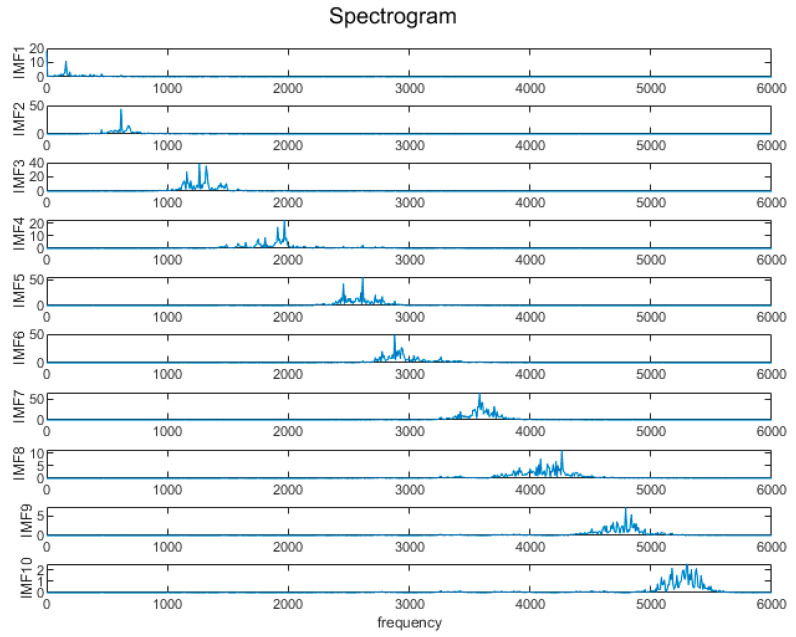
VMD decomposition spectrogram.

In Figure 6, u-1 denotes the optimal IMF component of the first fault type, and similarly, there exists a total of ten optimal IMF components. The *X*-axis represents the time domain, showing the changes in the signal at different time points, and the *Y*-axis represents the amplitude of the signal; that is, the signal strength at each time point. Figure 7 shows the IMF component spectrum diagram after optimizing VMD and decomposing it using combined entropy H as an indicator. It can be seen that the peak distinction between each component is obvious, and there is no signal aliasing. The signal is effectively decomposed during the signal decomposition process. The *X*-axis represents the frequency domain, showing the distribution of the signal at different frequency points, and the *Y*-axis represents the amplitude of the spectrum; that is, the strength of the signal at the corresponding frequency point.

### 4.4. Fault Diagnosis Result 1

**Figure 8:**
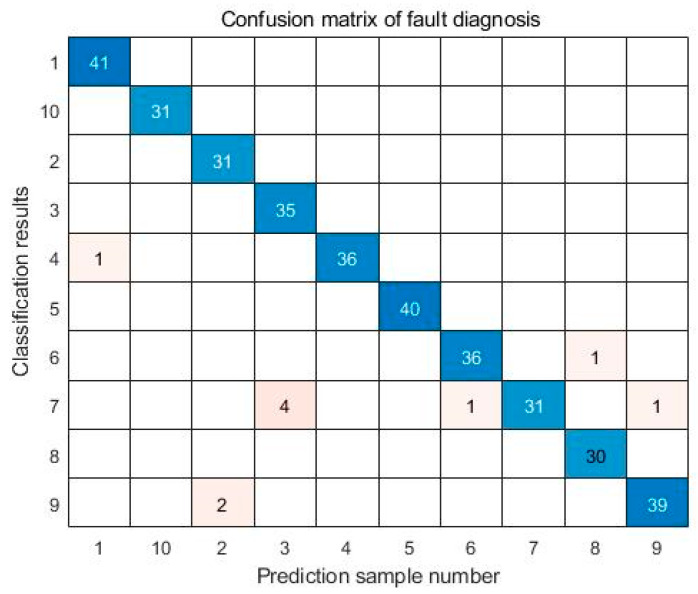
Confusion matrix of fault diagnosis 1.

**Figure 9:**
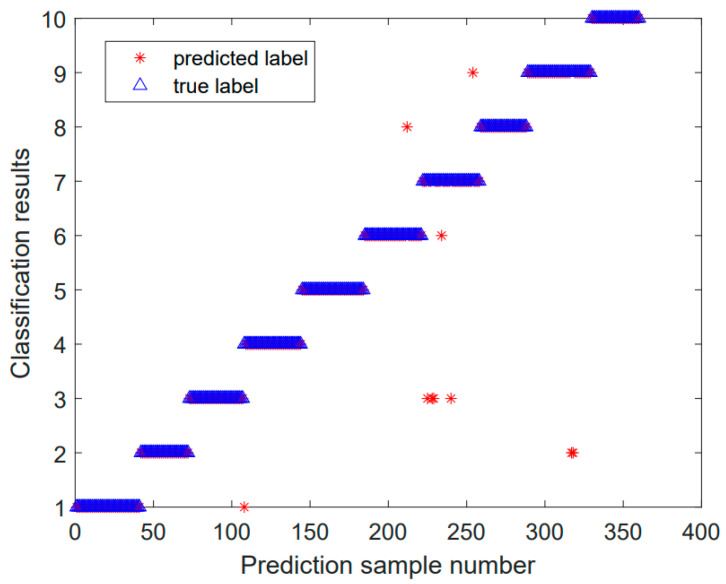
SABO–VMD–WMH–KNN fault diagnosis result 1.

**Figure 10:**
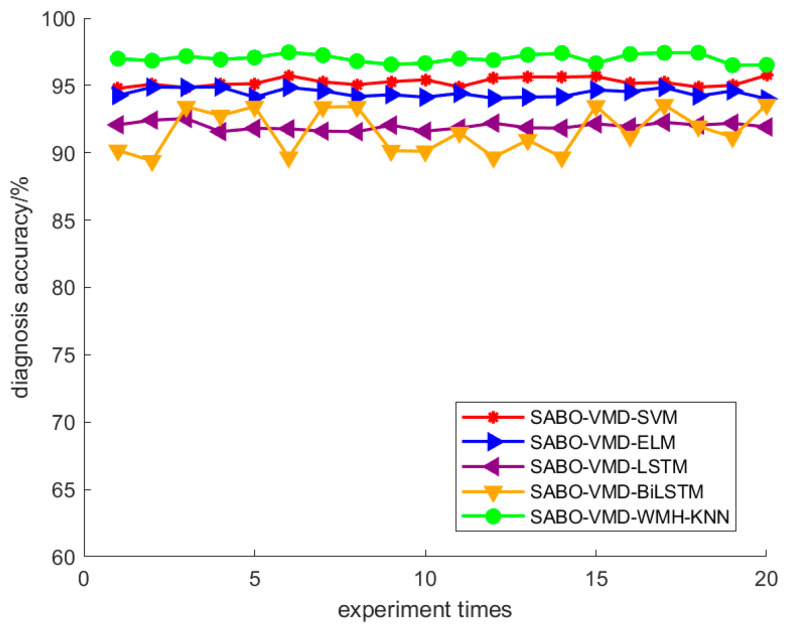
Fault diagnosis results of different methods.

**Figure 11:**
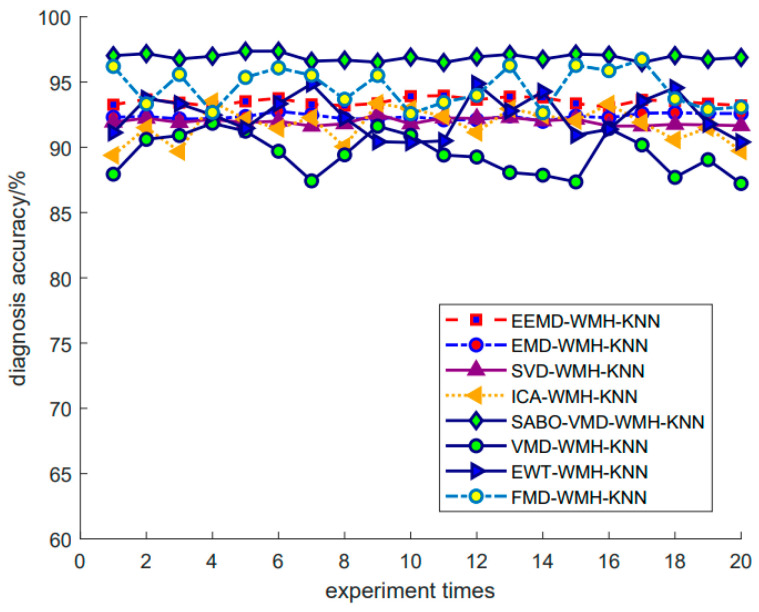
Fault diagnosis results of different signal decomposition methods.

Using the SABO–VMD–WMH–KNN diagnostic model, the diagnostic confusion matrix of ten faults is shown in Figure 8. The results of the SABO–VMD–WMH–KNN diagnosis are shown in Figure 9, while Figure 10 shows the diagnosis results of each diagnosis model running 20 times. The fault diagnosis result one outcomes are shown in Table 3. The diagnostic results of different signal decomposition methods are shown in Table 4. Figure 11 shows the diagnostic results after running 20 times of different signal decomposition diagnostic models.

As depicted in Table 3, the SABO–VMD–WMH–KNN diagnostic method exhibits the highest accuracy. Among them, the accuracy of the fault diagnosis of the SABO–VMD–KNN model that is not affected by WMH is significantly reduced. It can be seen that WMH–KNN is more adaptable to fault diagnosis classification. However, when compared to SABO–VMD–LSTM, it is noted that the diagnosis time is still relatively longer. Nevertheless, upon comprehensive consideration, the methodology presented in this article proves to be proficient in accurately and promptly identifying faults, boasting an impressive average diagnostic accuracy rate of 97.22%. In Table 4, the fault diagnosis outcomes obtained from the integration of five distinct signal decomposition methods with the WMH–KNN classifier, as proposed in this article, are presented. Notably, the results reveal that the implementation of the SABO–VMD signal decomposition model, as employed in this study, exhibits superior diagnostic accuracy when combined with the WMH–KNN classifier, emerging as the most accurate approach. In Figure 8, faults 1 to 10 correspond to T, IF-7, RF-7, OF-7, IF-14, RF-14, OF-14, IF-21, RF-21, and OF-21, representing ten distinct fault types. Notably, Figure 9 illustrates that, in terms of individual diagnosis, fault three (RF-7) exhibits the lowest accuracy compared to other fault types. Under heavy load conditions, the accuracy of the model decreases due to the nonlinear increase in the signal caused by load changes. However, the overall performance remains commendable. Figure 10 provides a visual representation of the diagnostic accuracy achieved by different methods (SABO–VMD–WMH–KNN, SABO–VMD–SVM, SABO–VMD–ELM, SABO–VMD–LSTM, and SABO–VMD–BiLITM) over 20 iterations. In comparison to the outlined method in this article (SABO–VMD–WMH–KNN), SABO–VMD–WMH–KNN consistently demonstrates the highest average diagnostic accuracy. Figure 11 intuitively illustrates the signal decomposition process using various methods combined with the WMH–KNN classifier (SABO–VMD–WMH–KNN, EEMD–WMH–KNN, EMD–WMH–KNN, SVD–WMH–KNN, ICA-WMH–KNN, EWT–WMH–KNN, and FMD–WMH–KNN). The diagnostic accuracy is obtained based on 20 iterations. Notably, SABO–VMD–WMH–KNN still exhibits the highest average diagnostic accuracy among the methods explored in this study. High accuracy and an F1 score indicate that the model is able to effectively distinguish different fault states. The long calculation time indicates that the optimization process is complex but the performance gains are significant.

From the above analysis, it can be seen that the SABO–VMD–WMH–KNN method proposed in this article shows the best performance in fault diagnosis compared with the baseline technology, which is mainly reflected in its excellent accuracy and ability to process complex signals. Specifically, the average diagnostic accuracy of this method reaches 97.22%, which is significantly higher than other methods, indicating its excellent performance in extracting and classifying fault features. Compared with SABO–VMD–KNN, which does not use WMH, WMH–KNN effectively handles the correlation between features through weighted Mahalanobis distance and improves classification accuracy. Although SABO–VMD–LSTM is shorter in calculation time, its accuracy is lower than SABO–VMD–WMH–KNN, which still maintains high accuracy even when dealing with complex load changes. In addition, SABO–VMD–WMH–KNN performs best among various signal decomposition methods. Although the calculation time is longer, its significant performance improvement proves its practical value in fault diagnosis. Taking into account accuracy and computational efficiency, this technology provides an excellent performance balance.

## 5. Experimental Case Analysis 2

### 5.1. Dataset Collection 2

**Figure 12:**
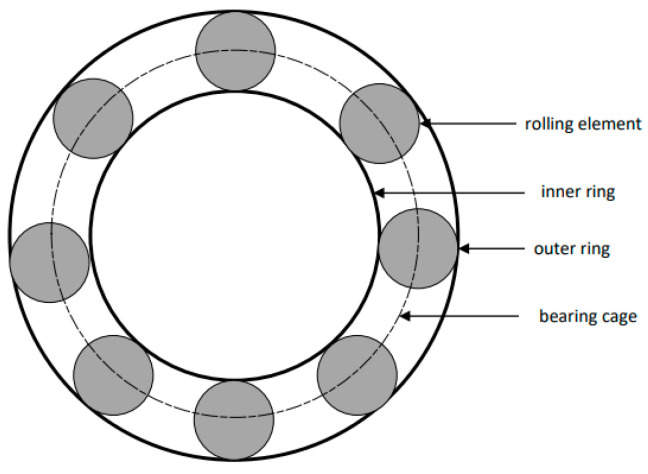
Various parts of bearings.

Experimental case two comes from the real-time operating data of the ZJ50DB electric drilling rig drawworks bearing. It uses vibration sensors and rotational speed sensors as sensing data to build a multi-category bear fault detection dataset. The dataset includes five different categories, namely normal status, inner ring failure, outer ring failure, rolling element failure, and cage failure. Each part of the bearing is shown in Figure 12.

The dataset contains 1000 independent data samples, which are divided into training sets and test sets in a ratio of 7:3 to ensure the independence of model training and evaluation. The training set contains a total of 700 data samples, including 500 samples in the normal state and 50 samples in each of the other four gear fault states. The test set contains a total of 300 data samples, including 208 samples in the normal state and 23 samples in each of the other four gear fault states.

### 5.2. Fault Diagnosis Result 2

With the same processing steps as experimental case one, after the VMD decomposition of Dataset 2, the optimal IMF component of each fault category is obtained, and then the WMH–KNN model is used for classification. The training and evaluation of the model use a variety of machine learning and deep learning algorithms for processing multi-category classification problems. Finally, the test set data are used to evaluate the performance of the model, including the accuracy and running time, to verify the effectiveness of the model in bearing fault detection tasks. Additionally, a confusion matrix analysis was performed to gain a more detailed understanding of the model’s classification performance.

**Figure 13:**
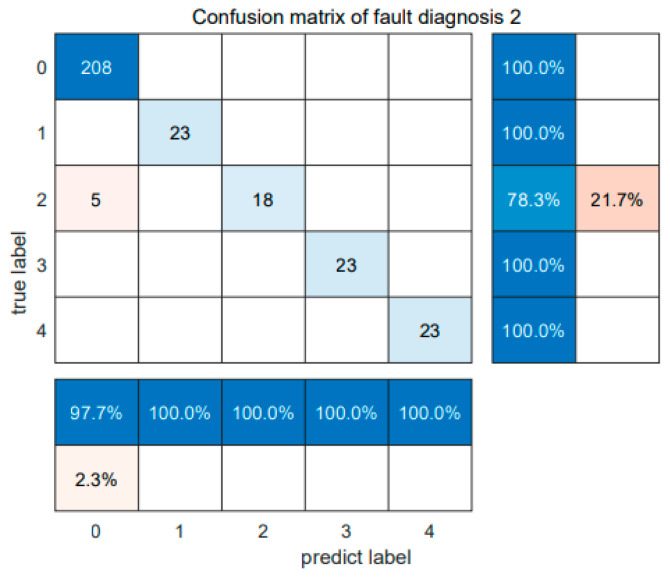
Confusion matrix of fault diagnosis 2.

**Figure 14:**
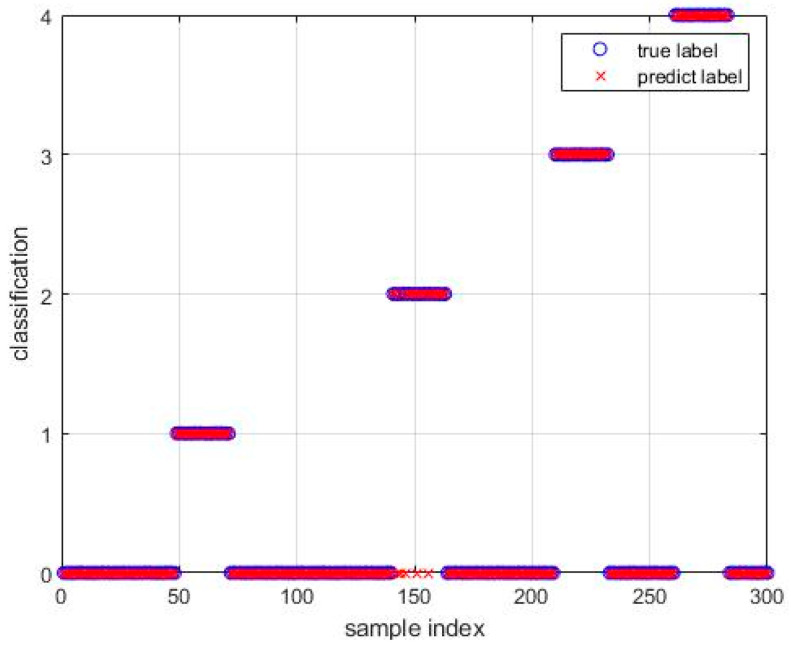
SABO–VMD–WMH–KNN fault diagnosis result 2.

**Figure 15:**
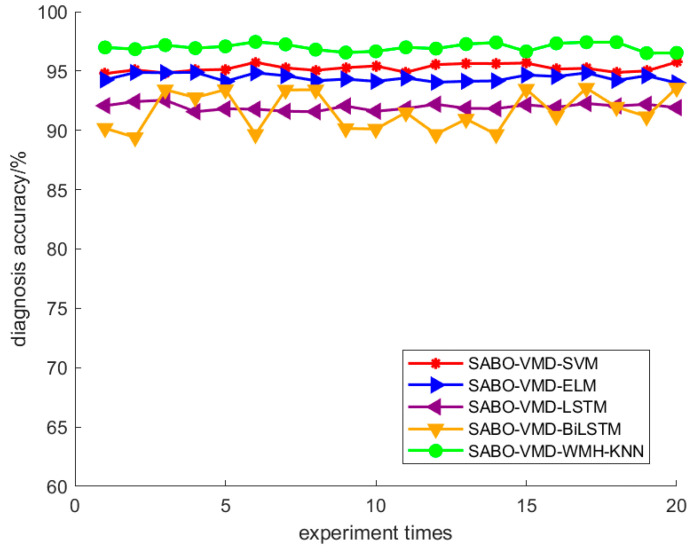
Fault diagnosis results of different methods 2.

**Figure 16:**
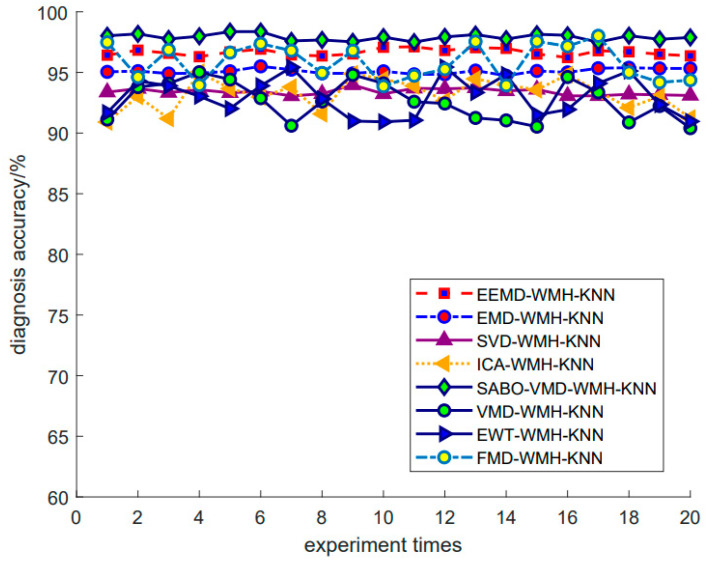
Fault diagnosis results of different signal decomposition methods 2.

In Figure 13, the diagnostic confusion matrix for the five faults in Dataset 2 is illustrated. Subsequently, Figure 14 displays the results of the SABO–VMD–WMH–KNN diagnostic model, while Figure 15 presents a comparative analysis of different diagnostic models. Each diagnostic model underwent 20 runs to ensure robustness, and the summarized fault diagnosis outcomes are presented in Table 5. Table 6 provides a comprehensive overview of the diagnostic results employing various signal decomposition methods. Additionally, Figure 16 visually represents the aggregated diagnostic outcomes after running each signal decomposition diagnostic model 20 times. This thorough evaluation allows for a detailed understanding of the performance and reliability of the SABO–VMD–WMH–KNN diagnostic model in comparison to other signal decomposition methods. The repetition of runs ensures the stability and consistency of the diagnostic results, contributing to a more comprehensive assessment of the model’s effectiveness.

As shown in Table 5, the SABO–VMD–WMH–KNN diagnosis method still shows the highest accuracy in Dataset 2. However, compared with EMD–WMH–KNN in signal decomposition, we note that the decomposition time is relatively long, but the diagnostic rate of this method is low because the EMD decomposition modes are easy to aliases. All things considered, the method proposed in this article can still accurately and timely identify faults in Dataset 2, with an average diagnosis accuracy of 98.33%. Table 6 lists the fault diagnosis results obtained by applying the five different signal decomposition methods proposed in this article to the WMH–KNN classifier on Dataset 2. The results showed that the SABO–VMD signal decomposition model employed in this study showed excellent diagnostic accuracy when used in conjunction with the WMH–KNN classifier, becoming the most accurate method. In Figure 13 and Figure 14, categories 0 to 4, respectively, correspond to five different fault types: normal status, inner ring failure, rolling element failure, and cage failure. It is worth noting that in terms of individual diagnosis, the normal state is recognized as having the lowest accuracy compared to other fault types. However, the overall performance is still commendable. Figure 15 visually shows the accuracy of 20 iterations of different methods (SABO–VMD–WMH–KNN, SABO–VMD–SVM, SABO–VMD–ELM, SABO–VMD–LSTM, and SABO–VMD–BiLITM). The method outlined in this paper (SABO–VMD–WMH–KNN) consistently showed the highest average diagnostic accuracy. Figure 16 visually illustrates the use of various methods combined with WMH–KNN classifiers (SABO–VMD–WMH–KNN, VMD–WMH–KNN, EEMD–WMH–KNN, EMD–WMH–KNN, SVD–WMH–KNN, ICA-WMH–KNN, EWT–WMH–KNN, and FMD–WMH–KNN). The diagnostic accuracy is obtained based on 20 iterations. Notably, SABO–VMD–WMH–KNN still exhibits the highest average diagnostic accuracy among the methods explored in this study. The accuracy and F1 score are slightly lower than the Case Western Reserve dataset but still perform well, verifying the generalization ability of the model. The calculation time is reduced, indicating that SABO is more efficient in optimizing different datasets.

The SABO–VMD–WMH–KNN method outperforms baseline techniques, particularly in its impressive accuracy and effective fault diagnosis capabilities. Achieving an average diagnostic accuracy of 98.33% in Dataset 2 significantly surpasses other decomposition methods, such as EMD–WMH–KNN, which suffer from lower accuracy due to pattern aliasing. Despite the longer signal decomposition time required by SABO–VMD, its combination with WMH–KNN—which leverages a weighted Mahalanobis distance—enhances classification accuracy well beyond that of traditional KNN methods. Overall, while the SABO–VMD–WMH–KNN method has a longer calculation time, its superior optimization and robust generalization make it the most effective choice for fault diagnosis across various datasets.

## 6. Experimental Case Analysis 3

### 6.1. Dataset Collection 3

Dataset 3 is the MPBFDD dataset, with a data acquisition frequency of 10 kHz, a single data length of 5 s, a digital vibration signal time series signal format, and a storage type of CSV file. This dataset collects the vibration signals of the mud pump bearing when the mud pump is running during a well operation, covering vibration signals under different working conditions. The dataset extracts a variety of fault types in the actual operating environment, which is used to verify the generalization ability of the model and help improve the reliability and accuracy of the fault detection algorithm. The working condition types mainly include load conditions, operating speeds, and environmental conditions. The load conditions include a light load, medium load, and heavy load; the operating speeds include a low speed, medium speed, and high speed; the environmental conditions include a normal temperature, low temperature, and high temperature. The fault types extracted from the dataset are a normal state, inner ring wear, outer ring wear, rolling element cracks, and cage damage. Five groups of data files were selected, including normal conditions, a light load, a low speed, and a normal temperature; an inner ring fault, a heavy load, a medium speed, and a high temperature; an outer ring fault, a medium load, a high speed, and a normal temperature; a rolling element fault, a light load, a low speed, and a high temperature; a cage fault, a heavy load, a medium speed, and a normal temperature.

Considering the diversity and sufficiency of each combination, 200 data were collected for each combination. In order to verify the effectiveness and robustness of the SABO–VMD–WMH–KNN method, this dataset not only uses the specific data of downhole mud pump operations but also improves the generalization ability of the dataset by reorganizing the dataset, thereby verifying the versatility of this method on different machines.

### 6.2. Fault Diagnosis Result 3

In this section, the results of the fault diagnosis using the third dataset are presented, focusing on the effectiveness of the SABO–VMD–WMH–KNN approach. The analysis aims to evaluate the performance of our proposed approach under various operating conditions, including a normal operation, inner race fault, outer race fault, rolling element fault, and cage fault. The test results of the proposed approach will also be compared with those obtained using more advanced baseline methods to fully evaluate the accuracy and robustness of the approach.

The SABO–VMD–WMH–KNN approach is applied and compared in detail with baseline methods such as deep convolutional neural networks (DCNNs), CNN-LSTM, and Transformer models, aiming to determine how the proposed approach performs compared to these state-of-the-art solutions. This detailed comparison will help highlight the strengths of our approach and potential areas for improvement, providing insights into its practical applicability and effectiveness for fault diagnosis in different scenarios.

**Figure 17:**
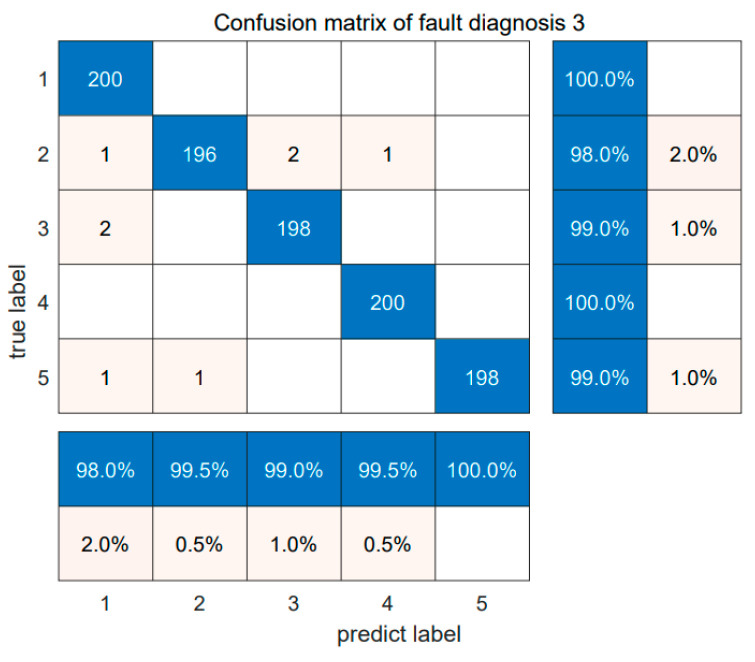
Confusion matrix of fault diagnosis 3.

**Figure 18:**
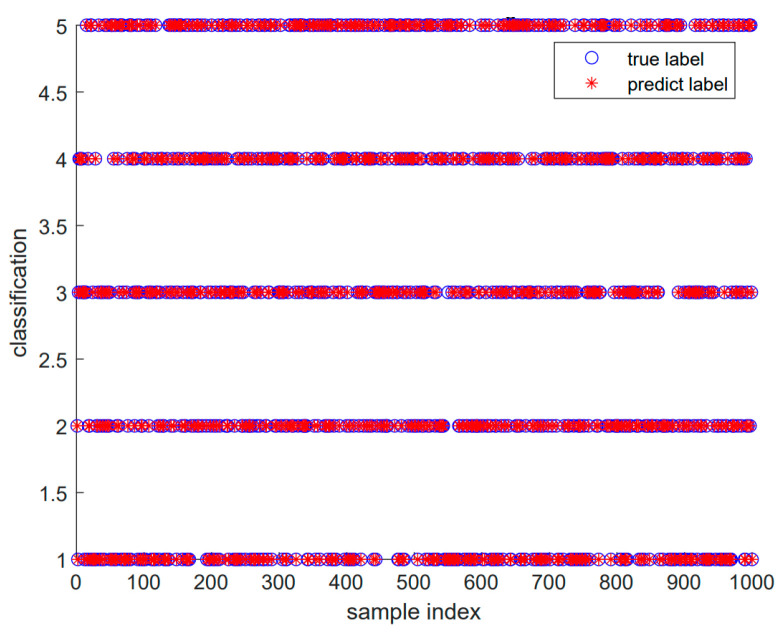
SABO–VMD–WMH–KNN fault diagnosis result 3.

**Figure 19:**
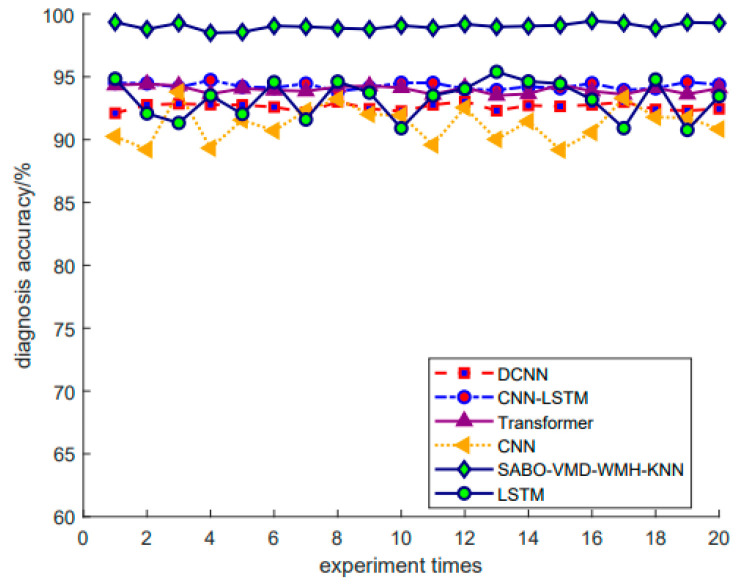
Fault diagnosis results of different methods 3.

The diagnosis results are shown below. Each diagnosis model was run 20 times to ensure robustness, and the summarized fault diagnosis results are shown in Table 7. Figure 17 shows the diagnosis confusion matrix of five faults in Dataset 3. Subsequently, Figure 18 shows the results of the SABO–VMD–WMH–KNN diagnosis model, while Figure 19 provides a comparative analysis of different diagnosis models. This comprehensive evaluation provides a detailed understanding of the performance and reliability of the SABO–VMD–WMH–KNN diagnosis model through detailed comparisons with other baseline methods.

As shown in Table 7, the SABO–VMD–WMH–KNN diagnostic method continues to demonstrate the highest diagnostic accuracy in Dataset 3, albeit with a relatively long fault diagnosis time compared to baseline methods such as DCNN and LSTM. While it outperforms DCNN and LSTM in terms of diagnostic time, the overall performance of these baseline methods does not match that of the proposed model. Considering all factors, SABO–VMD–WMH–KNN stands out as the best-performing model in terms of accuracy, making it suitable for tasks requiring extremely high accuracy.

CNN-LSTM and Transformer models offer high accuracy and are well-suited for processing complex time series data, though their computation times are longer. The LSTM model strikes a good balance between accuracy and computation time. However, the performance of DCNN and CNN is relatively low, indicating that they may be more appropriate for other specific tasks.

Therefore, the method proposed in this paper can still accurately and promptly identify faults in Dataset 3, achieving an average diagnostic accuracy of 99.2%. Testing across three datasets has demonstrated the ability of the proposed method to generalize well.

Figure 17 shows the fault diagnosis confusion matrix for the proposed method applied to Dataset 3. The method performs well in detecting the five types of data, especially for normal conditions: a light load, a low speed, a normal temperature, an inner ring fault, a heavy load, a medium speed, and high-temperature scenarios, achieving a diagnosis rate of 100%.

Figure 18 compares the fault diagnosis results with actual results, clearly illustrating the effectiveness of the SABO–VMD–WMH–KNN model under different fault types. The comparison results indicate that the model can accurately distinguish between normal states and various fault types, with diagnosis results highly consistent with actual conditions. The data points in Figure 18 further validate the significant advantage of the SABO–VMD–WMH–KNN model in terms of accuracy.

Figure 19 intuitively displays the accuracy of 20 iterations for different methods (SABO–VMD–WMH–KNN, CNN-LSTM, Transformer, LSTM, DCNN, CNN). As seen in the figure, SABO–VMD–WMH–KNN consistently maintains the highest accuracy across all iterations, with an average accuracy of 99.2%. In contrast, while the accuracy of other methods fluctuates, it generally remains lower than that of SABO–VMD–WMH–KNN. Among them, CNN-LSTM, Transformer, and LSTM perform relatively well but do not exceed 95% accuracy. DCNN and CNN perform relatively poorly, with accuracy fluctuating around 90%. These results further confirm the excellent performance and stability of the SABO–VMD–WMH–KNN method in fault diagnosis.

The model proposed in this paper, compared with benchmark methods through testing on Dataset 3, not only reflects its superior performance but also verifies its generalization ability and versatility across different machines.

## 7. Conclusions

In this paper, a motor-bearing fault diagnosis method based on SABO–VMD–WMH–KNN was proposed, which is capable of handling mixed vibration signals with multiple frequency components acquired under diverse operational conditions. The approach encompassed three key processes: firstly, employing SABO–VMD to decompose the fault signal into different modes and selecting the optimal mode for each fault type; secondly, utilizing the mean value, variance, peak value, and kurtosis of the optimal mode as feature vectors; thirdly, establishing the fault diagnosis model using the WMH–KNN method to execute the fault diagnosis process, making the characteristics of each fault type more obvious. The primary focus of this method was on addressing intricate working conditions where multiple frequency components are intertwined within vibration signals.

The main contribution of this paper is the introduction of the innovative SABO algorithm, which is applied to optimize VMD parameters and aims to overcome the performance limitations of traditional optimization algorithms in fault signal decomposition. Through SABO optimization, this paper successfully improves the global performance of VMD parameter optimization, making the decomposition of vibration signals more comprehensive and efficient and providing more accurate and powerful modal information for fault diagnosis. Furthermore, the article used WMH–KNN to make the KNN classification method more suitable for fault diagnosis and used weight factors to make the classification more accurate. Finally, the SABO–VMD–WMH–KNN comprehensive fault diagnosis model was formed by cleverly integrating the SABO-optimized VMD and the WMH–KNN method. This integration combined optimized parameter extraction with machine learning classification to provide a more comprehensive and efficient solution for electric drilling rig fault diagnosis.

Therefore, this method is anticipated to find extensive application in real industrial settings, enhancing the reliability, precision, and efficiency of fault diagnosis, thereby extending the longevity of equipment and systems, ultimately making a positive impact on the domain of industrial production. The method also has some limitations. The SABO–VMD–WMH–KNN method involves complex computational steps, including multiple iterations of VMD and calculation of Mahalanobis–Hamming distance, which may lead to a heavy computational burden when processing large-scale datasets. Especially for high-dimensional data and large datasets, the increase in computational complexity may affect the real-time and practical performance of the algorithm. Future research should be committed to optimizing the computational efficiency of the algorithm and exploring methods to reduce computational complexity to improve the robustness of the method.

## Figures and Tables

**Table 1:** Introduction of bearing dataset.

Bearing Failure Type	Label	Training Set Data	Test Set Data
T	1	840 × 2048	360 × 2048
IF-7	2	840 × 2048	360 × 2048
RF-7	3	840 × 2048	360 × 2048
OF-7	4	840 × 2048	360 × 2048
IF-14	5	840 × 2048	360 × 2048
RF-14	6	840 × 2048	360 × 2048
OF-14	7	840 × 2048	360 × 2048
IF-21	8	840 × 2048	360 × 2048
RF-21	9	840 × 2048	360 × 2048
OF-21	10	840 × 2048	360 × 2048

**Table 2:** Decomposition of optimal *K* and *α* values for each fault type.

Bearing Failure Type	K Value	α
T	8	1435
IF-7	6	645
RF-7	7	924
OF-7	7	675
IF-14	8	854
RF-14	6	1245
OF-14	9	832
IF-21	5	956
RF-21	7	1109
OF-21	8	984

**Table 3:** Fault diagnosis result 1.

Model	Average Accuracy (%)	Time (s)
SABO–VMD–WMH–KNN	97.22	1684.72
SABO–VMD–SVM	95.53	1698.42
SABO–VMD–ELM	94.72	1822.52
SABO–VMD–KNN	93.54	1634.35
SABO–VMD–LSTM	92.31	1582.34
SABO–VMD–BiLITM	91.24	1723.53

**Table 4:** Diagnostic results of different signal decomposition methods.

Model	Average Accuracy (%)	Time (s)
SABO–VMD–WMH–KNN	97.22	1684.72
FMD–WMH–KNN	94.37	1838.57
EEMD–WMH–KNN	93.73	1976.65
EMD–WMH–KNN	92.65	1735.73
EWT–WMH–KNN	92.58	1759.36
SVD–WMH–KNN	92.31	1656.76
ICA-WMH–KNN	91.24	1836.24
VMD–WMH–KNN	89.48	1535.67

**Table 5:** Fault diagnosis result 2.

Model	Average Accuracy (%)	Time (s)
SABO–VMD–WMH–KNN	98.33	1256.23
SABO–VMD–SVM	96.89	1378.56
SABO–VMD–ELM	95.39	1542.23
SABO–VMD–LSTM	93.78	1428.61
SABO–VMD–BiLITM	92.76	1563.89

**Table 6:** Diagnostic results of different signal decomposition methods 2.

Model	Average Accuracy (%)	Time (s)
SABO–VMD–WMH–KNN	98.33	1256.23
EEMD–WMH–KNN	95.69	1572.49
FMD–WMH–KNN	95.66	1325.64
EMD–WMH–KNN	94.38	1139.25
ICA-WMH–KNN	93.29	1286.46
EWT–WMH–KNN	93.14	1251.23
VMD–WMH–KNN	92.66	1123.89
SVD–WMH–KNN	91.59	1278.59

**Table 7:** Fault diagnosis result 3.

Model	Average Accuracy (%)	Time (s)
SABO–VMD–WMH–KNN	99.2	1962.85
DCNN	92.8	1897.28
CNN-LSTM	94.5	2016.43
Transformer	94.2	1987.46
CNN	91.5	1935.74
LSTM	93.2	1823.65

## Data Availability

Dataset 1 is a public dataset from Case Western Reserve University in the United States, and Dataset 2 is a dataset of a drilling rig in China. Due to privacy and confidentiality, it is not convenient to make it public. Dataset 3 is a mud pump dataset of a drilling platform in China. Due to copyright issues, it is not convenient to make it public.
